# Mechanism of macroalgae *Gracilaria bailiniae* responding to cadmium and lanthanum

**DOI:** 10.3389/fpls.2022.1076526

**Published:** 2022-12-01

**Authors:** Bowen Huang, Jianjun Cui, Yu Ran, Chunli Chen, Feng Li, Yulei Zhang, Zailiang Li, Enyi Xie

**Affiliations:** ^1^ Fishery College, Guangdong Ocean University, Zhanjiang, China; ^2^ Guangdong Laboratory of Marine Ecology Environment Monitoring and Warning, Zhanjiang, China

**Keywords:** cadmium, lanthanum, *Gracilaria bailiniae*, response mechanism, detoxication

## Abstract

Macroalgae can accumulate a wide array of metals, leading to their appliance as biomonitors of aquatic environments. With the rapid development of industrial and agricultural-based activities, Cd pollution in aquatic environments is considered an increasingly severe problem worldwide. Although La could alleviate the Cd stress in higher terrestrial plants, the response mechanisms of macroalgae to Cd and La are unknown. Along these lines, in this work, Cd significantly affected the growth, internal cellular structure, photosynthesis, pigment content, antioxidant enzyme activity, and lipid peroxidation level of *G. bailiniae*. However, the presence of La alleviated these adverse effects from Cd. Furthermore, the response mechanism of *G. bailiniae* to Cd was attributed to the self-antioxidant ability enhancement, membrane defense, and programmed-cellular regulation. However, the presence of La mediated the biosynthesis of both flavonoids and lipids, which inhibited the Cd accumulation, modulated algal stress signalling networks, renewed the impaired chlorophyll molecule, maintained the activity of the crucial enzyme, enhanced antioxidant ability, and maintained the stabilization of redox homeostasis, alleviating the adverse impact from Cd and improve the growth of *G. bailiniae*. The experimental results successfully demonstrate a new detoxicant to alleviate Cd stress, promoting a more comprehensive array of macroalgal applications.

## Introduction

Diminishing fossil energy resources, global warming, and the world food crisis are hot global issues. Algae has attracted wide attention from the scientific community and environmentalists alike as a potential renewable energy source, a candidate for carbon capture and sequestration, and a future food source (Bast, 2011). China is abundant in algal resources, with 3000 to 4000 species. Among these, approximately 50 species are economically important food macroalgae, which account for approximately 50% of the world’s total harvested macroalgae (Zeng et al., 2000; He et al., 2018). According to the 2021 China Fishery Statistical Yearbook, the 2020 cultivation area of macroalgae, such as *Saccharina*, *Undaria*, *Pyropia*, *Gracilaria*, and *Sargassum*, totalled approximately 142,000 ha. Interestingly, the output reached 2.615 million tonnes, accounting for 12.2% of China’s total marine production of aquaculture. Macroalgae *Gracilaria* species are widely distributed in warm waters and tropical habitats of the South China Sea (Yang et al., 2015). *Gracilaria* is a source of natural products, such as fatty acids, pigments, polysaccharides, and protein (Martinez and Padilla, 2016). Raw *Gracilaria* is also used as fertilizer, animal feed, and processed as medication (Martinez and Padilla, 2016). They also play an essential ecological role in the coastal ecological environment, such as during the removal of water eutrophication, mitigation of harmful algal blooms (HABs), and capture and sequestration of carbon (Han et al., 2013; Yang et al., 2015; Huang et al., 2022).

Heavy metal pollution is emerging as an annoying global environmental problem over the past decade (Gao, 2016). Industrial and agricultural activities cause a vast and abundant accumulation of heavy metals in the aquatic environment (Costa et al., 2020). On top of that, their high toxicity, bio-accumulation, and non-degradability would seriously hazard plant and human health (Zhang et al., 2016; Liang et al., 2018; Christophoridis et al., 2019). Cadmium (Cd), one of the most notorious heavy metals, has been recognized for its nonphysiological functions and high toxicity in algae (Zhu, 2010). In the work of Zhu et al. (2015), the natural abundance of macroalgae decreased with increasing levels of Cd. In the recent decade, macroalgae were also heavily impacted by Cd on the South China Sea coast (Zhu et al., 2011; Zhu et al., 2017). In addition, the toxic effects of Cd have been reported in two economically important food macroalgae, *Gracilaria lemaneiformis*, and *Hizikia fusiformis*. It was revealed that Cd could suppress the growth rate and the photosynthetic electron transfer, decrease the content of the photosynthetic pigments, change antioxidant enzyme activities, and cause lipid peroxidation in these macroalgae (Xia et al., 2004; Zhu et al., 2011; Zhu et al., 2017). However, very few studies have thoroughly investigated the underlying response mechanisms of marine macroalgae to Cd.

Besides heavy metals, rare earth elements (REEs) are considered emerging metals. REEs comprise the series of lanthanides (Ln), scandium (Sc), and yttrium (Y) (Pinto et al., 2020). China produces 90% of REEs and has nearly monopolized the global REEs market for many years (Binnemans et al., 2013). Rapid technological advances make REEs nearly irreplaceable components of various electronic technologies. Exploiting these elements has increased their concentrations in soil and water (Binnemans et al., 2013; Costa et al., 2020). Furthermore, the recycling of REE was rarely applied recently due to inefficient techniques (Binnemans et al., 2021). Lanthanum (La) is the first element of Ln, and macroalgae have the highest concentration of La in marine organisms (Hou and Yan, 1998; Holden and Coplen, 2004; Herrmann et al., 2016). Few studies have investigated the influence of La on marine macroalgae (Herrmann et al., 2016; Figueiredo et al., 2022). Hence, further work on the marine macroalgae response mechanisms to La is still necessary.

Further, research by He et al. (2005) and Wang et al. (2012) reported that low concentrations of La could alleviate the Cd stress on the growth and photosynthetic function of higher terrestrial plants. The ability of La to facilitate plant recovery while under Cd stress might be attributed to the decrease in Cd absorption and accumulation. *Gracilaria bailiniae* (*G. bailiniae*), which is regarded as a heat-resisting species of *Gracilaria*, could survive in China’s southern provinces for around a year. It plays a vital role in the marine ecological environment, such as wastewater treatment and eco-environmental modification (Xu et al., 2021; Huang et al., 2022). Considering the alleviation of La addition in the higher terrestrial plants under Cd stress, we hypothesized that La could cross-talk with Cd in the *G. bailiniae*. To systematically investigate the metal response of *G. bailiniae*, the Cd and La effects on its growth and physiology are assessed, and response mechanisms through metabonomics are further revealed. The data outcomes should provide a solid theoretical basis for broad applications of *G. bailiniae* in aquaculture, wastewater treatment, and eco-environmental modification.

## Materials and methods

### Collection and pre-treatment of *G. bailiniae* material

400 g *G. bailiniae* were collected from Wushi bay (20°33′57″N, 109°49′57″E). The periphyton and the surface impurities of the *G. bailiniae* thalli were removed using sterilized seawater and a soft brush. The *G. bailiniae* thalli were then divided and precultured in ten 2-L laboratory glass containers (20 g L^−1^) containing 2 L of filter-sterilized (0.22-μm) natural seawater and enriched with f/2 medium without ethylenediaminetetraacetic acid (EDTA), (the initial pH was adjusted to the value of 8.0 with 0.1 mol·L^−1^ HCl or NaOH). All laboratory glassware was sterilised at a temperature of 121°C for 20 minutes in an autoclave steriliser to reduce bacterial numbers. EDTA would entirely decrease the toxicity of the heavy metals and REEs due to the chelating properties (Tai et al., 2010). The f/2 media contained all the essential and trace elements necessary for algae growth (Sun et al., 2019). The *G. bailiniae* were illuminated with fluorescent light at a light intensity of 6,000 lx using a 12:12-h light: dark cycle in a constant-temperature incubator at the temperature value of 33 ± 1°C, and a salinity of 30 PSU. This temporary culture period was 3–5 days to stabilize the physiological state of the *G. bailiniae* and enable the experiment.

### Growth assays of *G. bailiniae* exposed to Cd, La, and their combination

Standard cadmium chloride (99.99%, CAS number: 10108-64-2) and lanthanum nitrate (99%, CAS number: 10277-43-7) were purchased from Macklin Biochemical Technology Co., Ltd. (Shanghai, China). Five hundred mg cadmium chloride and 800 mg lanthanum nitrate were transferred into two 1-L conical flasks containing 1 L of the same f/2 medium, respectively. The resulting standard solutions (500 mg·L^−1^ Cd and 800 mg·L^−1^ La) were stored at the temperature value of 4°C. Seventy-two g intact *G. bailiniae* thalli of preculture were selected, divided, and added to twelve 1-L conical flasks, and also inoculated 1 L of the same f/2 medium (6 g L^−1^), respectively. After this step, the standard solutions of Cd and La were diluted to the groups to obtain final concentrations of 5 mg·L^−1^ Cd group, 8 mg·L^−1^ La group, and 5 mg·L^−1^ Cd + 8 mg·L^−1^ La group, respectively. From previous studies, these concentrations of Cd and La are in the moderate concentration range and could aptly evoke an extensive physiological and metabolic response in algae and higher plants (Babula et al., 2015; Kováčik et al., 2018; Shen et al., 2018). For the control groups, no Cd or La standard solutions were added. Three replicates for all groups were prepared in the experiment, with identical culture conditions as those of the *G. bailiniae* preculture, and lasted for seven days. In addition, the f/2 medium and metal element (Cd and La) were renewed every 2 d to simulate the same procedure of *G. bailiniae* exposed to these elements. After 7 d of exposure, the thalli were harvested, and the fresh weights (FW) of *G. bailiniae* were weighed. The relative growth rate (RGR) of *G. bailiniae* was determined by using equation (1):


(1) $$RGR(\%day^{-1})=\frac{\ln(W_{t}/W_{0})}{t}\times 100$$

where *W_0_* is the initial FW, and *W_t_* represents the FW after t days.

### Cd and La content analysis of *G. bailiniae*

The inductively Coupled Plasma Mass Spectrometry (ICP-MS) method was used to measure the Cd and La content of *G. bailiniae*. The working principle of the ICP-MS method is that the detected elements enter into a high-frequency plasma state through a particular form and become ionized at high temperature values. Afterward, the generated ions are focused by the ion optical lens and enter the quadrupole mass spectrometer to be separated according to the charge-to-mass ratio. The ICP-MS technique is a rapid, accurate, and efficient way to detect various trace elements with low detection limits and high sensitivity (Liu et al., 2021). The sample pre-treatment process followed the method of Cai et al. (2019). At the end of the experiment, 1 g *G. bailiniae* thalli was washed three times with ultrapure water, then dried and crushed in a constant temperature oven at 60°C for 12 h. Afterward, G. bailiniae dry powder was washed with a certain amount of nitric acid in a centrifuge tube. The washing solution was transferred into a digestion tank and heated to nitrolysis until dry. After the cooling step, ultrapure water was added to a final 15 mL volume, and the resulting solution was used to detect the Cd and La content in *G. bailiniae*. The “GB5009.268-2016-National Food Safety Standard-Determination method was used to analyse and determine quantities.

### Chlorophyll fluorescence of *G. bailiniae*

The parameter Fv/Fm, which refers to the maximum photochemical efficiency of photosystem II (PS II), was applied to describe the photosynthetic efficiency. Following dark-acclimation for 15 min, 0.1 g of *G. bailiniae* thalli was applied to determine values of Fv/Fm using a Hansatech FMS-2 modulated fluorometer (Hansatech Instruments, Norfolk, UK).

### Ultrastructure analysis of *G. bailiniae* exposed to Cd, La, and their combination

0.1 g thalli of *G. bailiniae* exposed to Cd, La, and their combination was washed three times using 0.1-M phosphate-buffered saline. Subsequently, the thalli were fixed for 12 h using 2.5% glutaraldehyde (stored at a temperature of 4°C overnight). Subsequently, the samples were embedded in the epoxy resin. The sections of thalli were made by utilizing a microtome (Leica EM UC7, Wetzlar, Germany), followed by staining with uranyl acetate and lead citrate for 20 min. Finally, the sections were observed using Transmission Electron Microscopy (TEM) (JEM-1400, Hitachi, Japan) measurements.

### Pigment content analysis of *G. bailiniae* exposed to Cd, La, and their combination

Aliquots of 0.1 g thalli of *G. bailiniae* were exposed to Cd, La, and their combination was collected and crushed after seven days. Subsequently, the pigment of chlorophyll a (chl a) and carotenoid of *G. bailiniae* were extracted from the crushed thalli using 5 mL 95% ethyl alcohol and analysed using ultraviolet spectrophotometry (Shimadzu UV2450, Shimane, Japan) at wavelengths of 470, 649, and 665 nm according to the method described by Wang et al. (2020). The pigment content was calculated using the following equations:


(2) $$chla\left(mg\cdot g^{-1}\right)=\left(13.95\times A_{665}-6.88\times A_{649}\right)\;V/M/1000$$

(3) $$carotenoid\left(mg\cdot g^{-1}\right)=\left(4.08\times A_{470}-11.56\times A_{649}+3.29\times A_{665}\right)\;V/M/1000$$

where A_470_, A_649_, and A_665_ denote the average optical density at 470, 649, and 665 nm, respectively, V stands for the volume of 95% ethyl alcohol (mL), M is the quality of *G. bailiniae* (g), and the unit of pigment content is mg·g^–1^.

The Phycoerythrin (PE) and phycocyanin (PC) contents were estimated according to the method reported by Beer and Eshel (1985). More specifically, 0.1 g thalli of *G. bailiniae* were crushed in 5 mL of 0.1 M phosphate buffer (pH 6.8), rinsed with 5 mL of buffer, and then centrifuged at 1000 × g for 10 min. Phycobiliprotein contents in the supernatant were calculated by using the following formulae:


(4) $$PE\left(mg\cdot g^{-1}\right)=\frac{\left[\left(A_{564}-A_{592}\right)-\left(A_{455}-A_{592}\right)\times 0.20\right]\times 0.12}{FW}$$

(5) $$PC\left(mg\cdot g^{-1}\right)=\frac{\left[\left(A_{618}-A_{645}\right)-\left(A_{595}-A_{645}\right)\times 0.51\right]\times 0.15}{FW}$$

where A_455_, A_564_, A_592_, A_595_, A_618_, and A_645_ are the average optical density at the wavelength numbers 455, 564, 592, 595, 618, and 645 nm, respectively, and FW is the fresh weights of *G. bailiniae*.

### The antioxidant enzyme activity and lipid peroxidation level analysis of *G. bailiniae* exposed to Cd, La, and their combination

Superoxide dismutase (SOD), peroxidase (POD), and catalase (CAT) belong to the antioxidant enzyme system. Malondialdehyde (MDA) is considered the most common lipid peroxidation marker. The four enzyme markers indicate the extent of algal cell oxidative stress and damage (Sun et al., 2019; Zheng et al., 2021). Following the manufacturer’s instructions, these indexes were determined using several kits (Shanghai Enzyme-linked Biotechnology Co., Shanghai, China). The enzyme activities were analysed and expressed as protein levels. Protein concentrations were determined using bicinchoninic acid (BCA) as the detection reagent for Cu^+^ following the reduction of Cu^2+^ by protein in an alkaline environment (BCA protein kit, Sangon company, China). Measurements were made using a microplate reader using a microwell plate protocol A590 in the manufacturer’s instructions.

### Metabolomics analysis of *G. bailiniae* exposed to Cd, La, and their combination

1 g *G. bailiniae* was exposed to control, Cd, La, and their combination systems (three replicates per group) and then weighed and frozen in liquid nitrogen. The sample preparation for the metabonomic analysis and data analysis were performed using standard procedures, and three biological replicates of each treatment were analysed. Ultra-performance liquid chromatography (UPLC) (SHIMADZU Nexera X2), in combination with tandem mass spectrometry (MS/MS) (Applied Biosystems 4500 QTRAP), was employed for data acquisition. LIT and triple quadrupole (QQQ) scans were also obtained on a triple quadrupole linear ion trap mass spectrometer (QTRAP) and AB4500 Q TRAP UPLC/MS/MS system. By using the self-built database MWDB (Metware database), the substances were qualitatively analysed according to the secondary spectrum information, and the multiple reaction monitoring mode (MRM) of the triple quadrupole mass spectrometer was used to quantify metabolites (Meng et al., 2022). A principal component analysis, an orthogonal partial least squares discriminant analysis model, and differentially accumulated metabolites (DAMs) were conducted and screened with |Log_2_FC (fold change)| ≥ 1, VIP (variable importance in the project) ≥ 1 and p values (Student’s t-test) ≤ 0.05. Finally, the KEGG database was used for the pathway enrichment analysis of DAMs.

### Statistical analysis

The normality and homogeneity of all data variances were tested using a Kolmogorov-Smirnov and a Levene test, respectively. To determine the impact of Cd and La, a one-way analysis of variance (ANOVA) was applied using IBM SPSS Statistics 25. Additionally, Duncan’s *post hoc* pairwise comparisons were used when all variances were equal. Otherwise, Welch’s ANOVA test was utilized. The Student’s t-test was conducted to assess significant differences. The data comparisons were deemed to have a significant difference at an alpha value of *P*< 0.05. Finally, the data visualizations were achieved using GraphPad Prism 7.

## Results

### Impact of Cd, La, and their combination on the growth and accumulation of *G. bailiniae*

**Figure 1:**
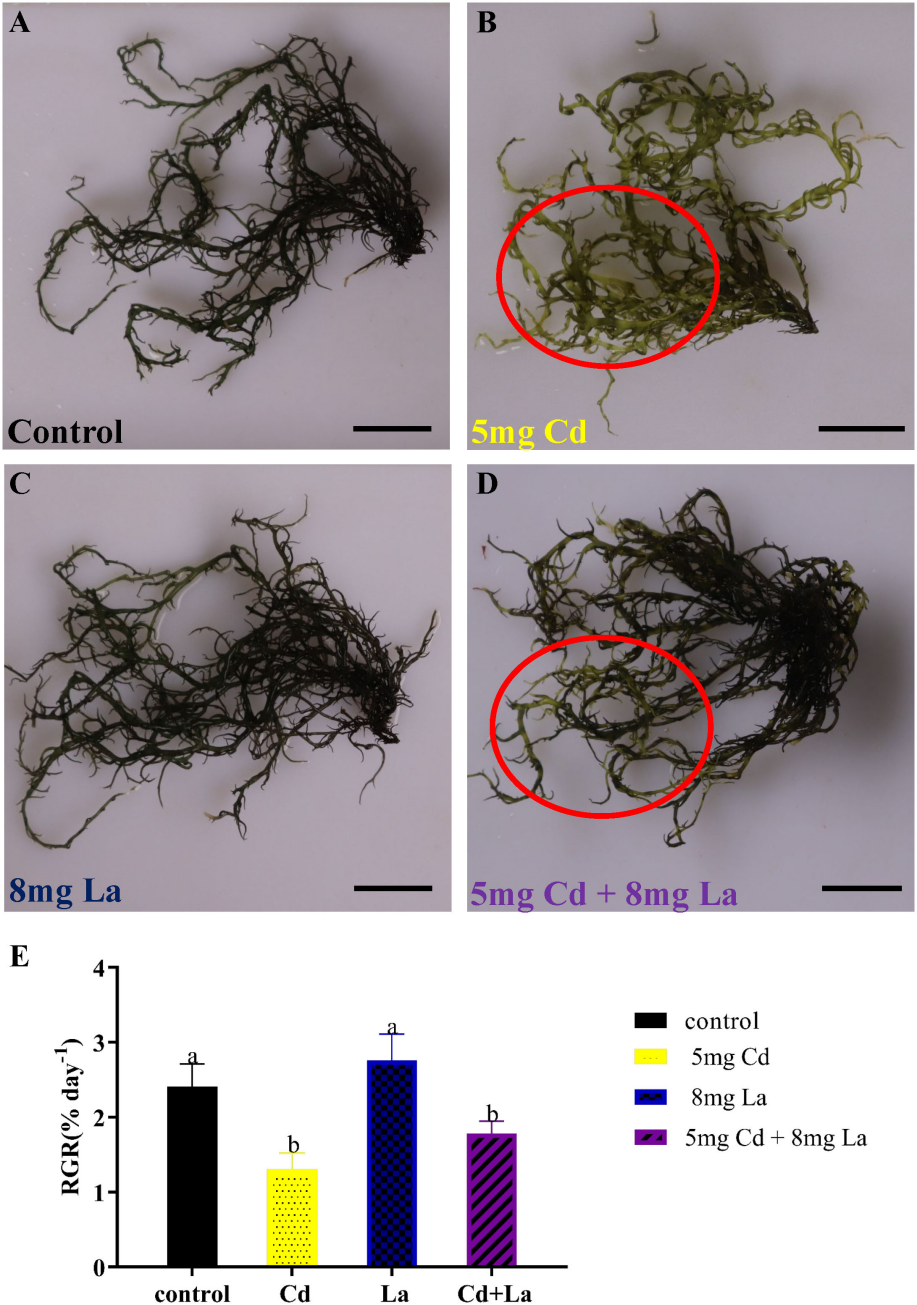
Impact of Cd, La, and their combination on *G. bailiniae*: **(A–D)** morphological changes, and **(E)** relative growth rate (RGR). Units: mg L^−1^. Red circles denote the discoloration of *G. bailiniae*. The scale bar represents 2 cm. The RGR data are presented as the mean ± standard deviation (n = 3). The lowercase letters indicate significant differences (ANOVA/Duncan ANOVA, *P<* 0.05).

From the morphology perspective, 5 mg·L^−1^ Cd treatment significantly discoloured the *G. bailiniae* thalli compared to the control samples (**Figures 1A, B**). Although 8 mg·L^−1^ La treatment displayed a limited effect on the *G. bailiniae* thalli (**Figures 1A, C**), the presence of La significantly alleviated the discolouration of *G. bailiniae* thalli exposed to Cd (**Figures 1B, D**). Regarding RGR, except for 8 mg·L^−1^ La, *G. bailiniae* was significantly affected by the different treatments compared to the control after seven days (**Figure 1E**) (*P*< 0.05). *G. bailiniae* growth exposed to 5 mg·L^−1^ Cd was significantly weaker than without imposing the Cd treatment, and RGR data decreased by 52.1%. However, 8 mg·L^−1^ La treatment slightly increased the *G. bailiniae* growth, with RGR increasing by 14.6%. As shown in Cd and La combination treatments, La presence improved the *G. bailiniae* growth under the application of the Cd treatment. Although these levels of *G. bailiniae* growth were still significantly suppressed compared to the control samples(RGR decreased by 34.5%), the RGR increased by 36.8% compared to the Cd treatment. Finally, the presence of La decreased the Cd accumulation in the thalli of *G. bailiniae* (see **Table 1**) (*P*< 0.05).

**Table 1:** Cd and La accumulated concentrations in the thalli of *G. bailiniae*.

	control	Cd	La	Cd + La
Cd concentration (mg g^−1^)	nd	4.57 ± 0.039a	nd	3.95 ± 0.076b
La concentration (mg g^−1^)	nd	nd	1.53 ± 0.040a	0.431 ± 0.0037b

The data are presented as the mean ± standard deviation (n = 3). The lowercase letters indicate significant differences (ANOVA/Duncan ANOVA, P< 0.05). ‘nd’ indicates ‘not detected’.

### Influence of Cd, La, and their combination on the chlorophyll fluorescence of *G. bailiniae*

**Figure 2:**
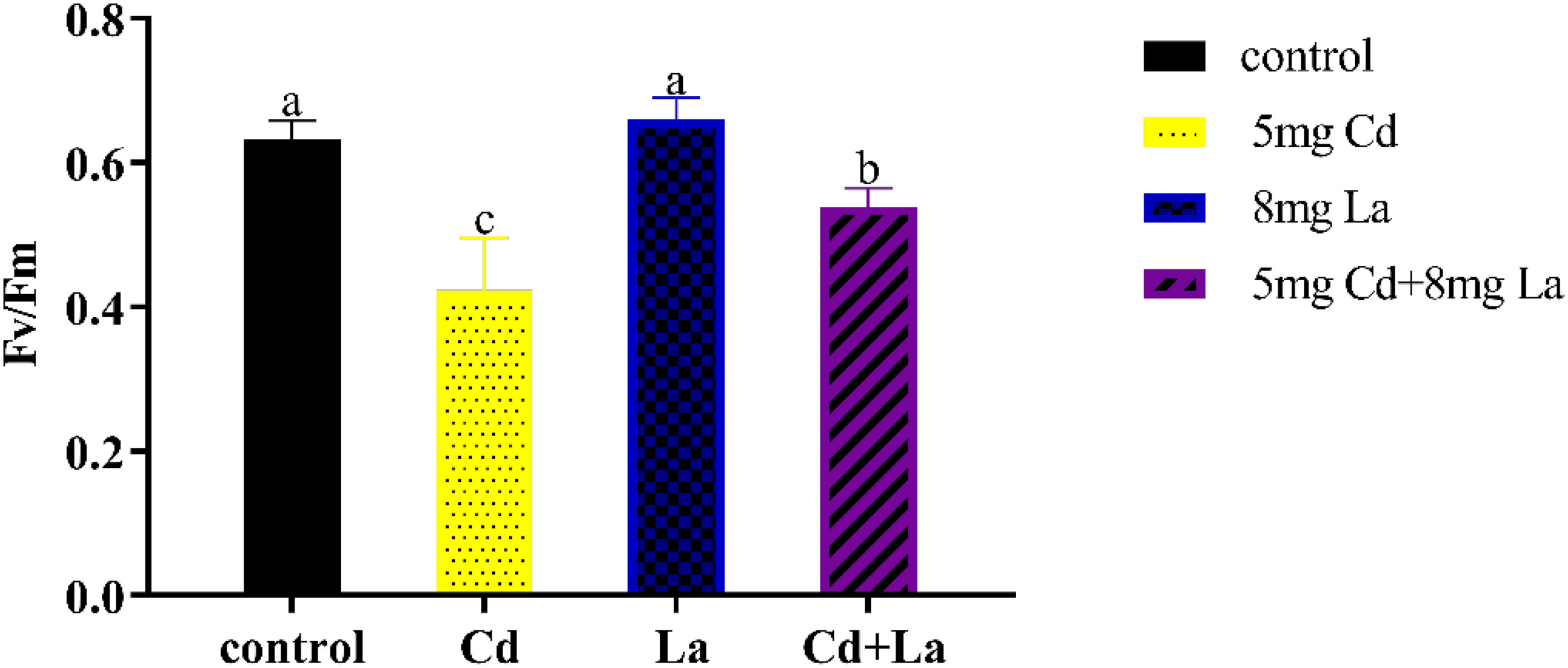
Photosynthetic efficiency (Fv/Fm, the maximum quantum yield of photosystem II) of *G. bailiniae* exposed to Cd, La, and their combination. Units: mg L^−1^. The data are presented as the mean ± standard deviation (n = 3). The lowercase letters indicate significant differences (ANOVA/Duncan ANOVA, *P<* 0.05).

Fv/Fm, which describes the maximum photochemical efficiency of PSII, represents the photosynthetic system’s photosynthetic efficiency. Under this direction, after being exposed to different treatments for 7 d, the acquired Fv/Fm values in *G. bailiniae* displayed various changes compared to the control group (**Figure 2**). The photosynthetic efficiency of 5 mg·L^−1^ Cd treatment was significantly lower than in those samples without the implementation of the Cd treatment (*P*< 0.05), and the Fv/Fm decreases 33.0%. The photosynthetic efficiency of the 8 mg·L^−1^ La treatment group displayed no significant difference from the control group (*P* > 0.05) but still increased by 4.32%. In addition, the presence of La improved the photosynthetic efficiency in Cd and La combination treatment, and its Fv/Fm increased by 27.3% compared to the Cd treatment. The photosynthetic efficiency, however, was still lower than the control group (*P* > 0.05) since the Fv/Fm decreased by 14.7%.

### Impact of Cd, La, and their combination on the ultrastructure of *G. bailiniae*

**Figure 3:**
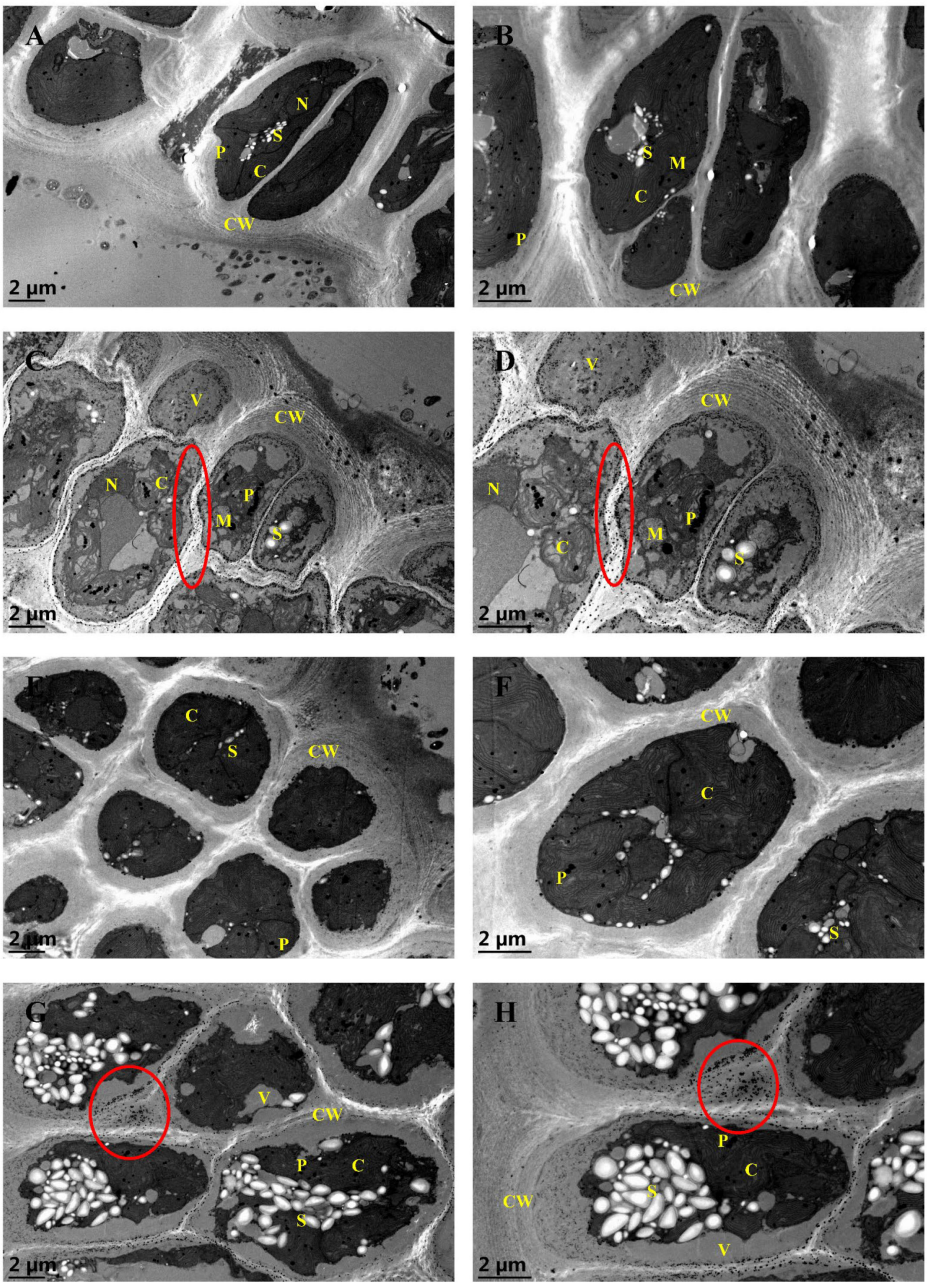
TEM ultrastructural images of *G bailiniae* cortical cells in control and treatment: **(A)** 8000x control, **(B)** 12000x control, **(C)** 8000x Cd treatment, **(D)** 12000x Cd treatment, **(E)** 8000x La treatment, **(F)** 12000x La treatment, **(G)** 8000x Cd + La treatment, **(H)** 12000x Cd + La treatment. Circles denote metal deposits; C, chloroplast; CW, cell wall; M, mitochondrion; N, nuclei; P, plastoglobuli; S, starch grain, and V, vacuole.

To examine the variations in the endocellular physiology of *G. bailiniae* cortical cells exposed to different treatments (control, 5 mg·L^−1^ Cd, 8 mg·L^−1^ La, and their combination treatment), valuable insights were derived from the comparison of the TEM images (**Figures 3A–H**). In the control group (**Figures 3A, B**), chloroplasts appeared intact, with an unstacked internal organization. Additionally, small mitochondria were present in association with the chloroplasts. Plastoglobuli, the electron-dense lipid droplets, were scattered between the thylakoids. A few starch grains were also stacked between chloroplasts in a perinuclear position. Nuclei and chromatin were diffused, and a legible and well-developed nucleolus was always present. Cortical cells were surrounded by a thick cell wall, where microfibrils had different electron densities and were embedded in an amorphous matrix consisting of sulphated polysaccharides. Concerning the 5 mg·L^−1^ Cd treatment group (**Figures 3C, D**), cortical cells appeared vacuolated by increasing the cell wall thickness, exhibiting concentric layers of microfibrils. The chloroplasts were degenerated, whereas plastoglobuli numbers increased and stacked in the disrupted chloroplasts. Small mitochondria were disrupted and swollen. The number of starch grains was reduced, and part of them was swollen. Numerous electron-dense precipitates were also present in the intercellular space, presumably Cd deposits. However, nuclei showed few changes in the ultrastructural organization. During the 8 mg·L^−1^ La treatment (**Figures 3E, F**), cortical cells revealed few changes in the ultrastructural organization, and no significant metal deposits were found in the intercellular space. During the implementation of the Cd and La combination treatment (**Figures 3G, H**), starch grains were significantly increased in numbers and swollen, stacking in the cortical cells. Compared to the Cd treatment, chloroplasts appeared more intact and well-shaped under the Cd and La combination treatment. Finally, cellular vacuolation, plastoglobuli stack, and concentric layers of microfibrils were reduced compared to the Cd treatment. Nonetheless, the metal deposits and increased cell wall thickness were still present.

### Influence of Cd, La, and their combination on the pigment content of *G. bailiniae*

**Figure 4:**
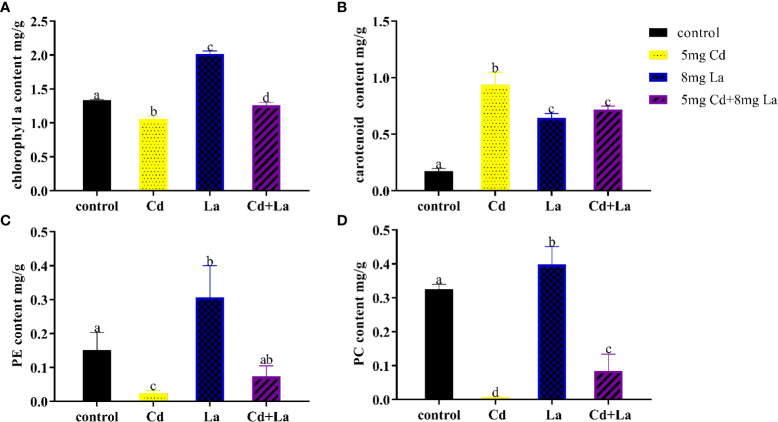
Pigment content of *G bailiniae* exposed to Cd, La, and their combination: **(A)** chl a, **(B)** carotenoid, **(C)** PE, **(D)** PC. Units: mg L^−1^. The data are presented as the mean ± standard deviation (n = 3). The lowercase letters indicate significant differences (ANOVA/Duncan ANOVA, *P*< 0.05).

Cd, La, and their combination were applied to explore the impact on the pigment content of *G. bailiniae* over seven days (**Figure 4**). Overall, the various treatments of these metals had a significant influence on the content of chl a, carotenoid, PE, and PC, respectively (*P*< 0.05). The chl a content at 5 mg·L^−1^ Cd, and the Cd and La combination treatments were significantly lower than the control group, with their content decreasing by 20.8% and 5.70%, respectively (**Figure 4A**) (*P*< 0.05). The chl a content of the 8 mg·L^−1^ La treatment was significantly higher than the control, and its content increased by 51.0% (**Figure 4A**) (*P*< 0.05). As can be observed from **Figure 4B**, Cd, La, and their combination treatment significantly increased the carotenoid content by 439%, 271%, and 313% compared to the control, respectively (*P*< 0.05). The pigment content of PE and PC also displayed a similar tendency when exposed to Cd, La, as well as the Cd and La combination treatment (**Figures 4C, D**). 5 mg·L^−1^ Cd, and Cd and La combination treatments significantly decreased the content of both PE and PC (*P*< 0.05). However, implementing the 8 mg·L^−1^ La treatment significantly increased the PE and PC contents (*P*< 0.05). In addition, although the PE and PC content of the Cd and La combination treatment was significantly decreased compared to the control, they were higher than during the Cd treatment (*P*< 0.05).

### Impact of Cd, La, and their combination on the antioxidant enzyme activity and lipid peroxidation level of *G. bailiniae*

Cd, La, and their combination were applied to explore their treatment outcomes on antioxidant enzyme activities and the lipid peroxidation of *G. bailiniae* over seven days (**Figure S1**). Except for CAT, 5 mg·L^−1^ of Cd treatment significantly increased other antioxidant enzyme activity. As shown in **Figure S1A**, the SOD activity is 66.0% higher than in the control group (*P*< 0.05). As is shown in **Figure S1B**, the POD activity is 39.7% higher than the control (*P*< 0.05), and the CAT activity is 16.5% higher than the control group (**Figure S1C**) (*P* > 0.05). In addition, applying the 5 mg·L^−1^ Cd treatment also significantly increased lipid peroxidation. The data in **Figure S1D** illustrates that the MDA content is 22.5% higher than in the control samples (*P*< 0.05). Compared to the control group, 8 mg·L^−1^ La treatment displayed a slight increase in the activity of both SOD and POD, and a limited effect on the MDA content (**Figure S1**) (*P* > 0.05). As shown in Cd and La combination treatment data, the presence of La alleviated the impact of Cd on the antioxidant enzyme system and lipid peroxidation levels. Finally, the antioxidant enzyme activity of SOD, POD, and CAT, and lipid peroxidation level (MDA content) of Cd and La combination treatment is 11.3%, 19.6%, 2.6%, and 13.8% lower than Cd treatment alone, respectively (**Figure S1**).

### Influence of Cd, La, and their combination on *G. bailiniae* metabolites

**Figure 5:**
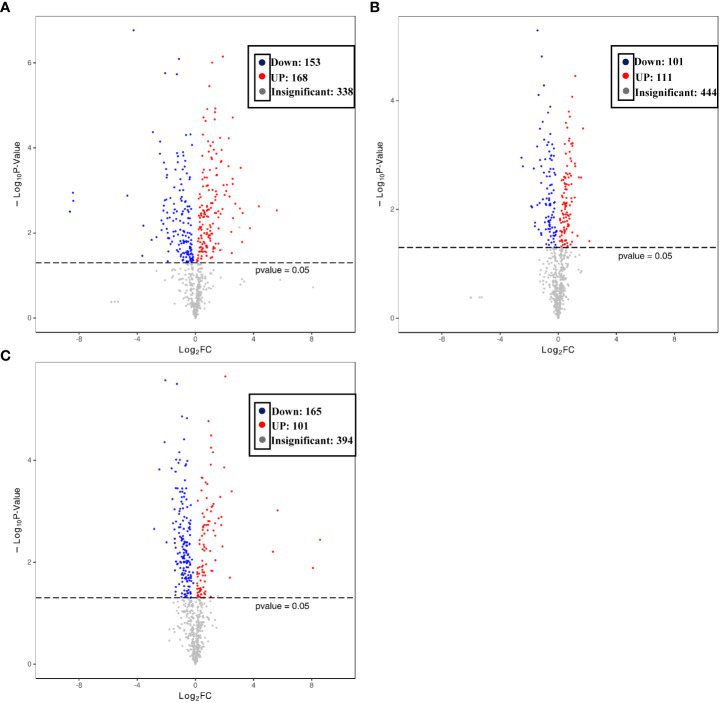
Volcano plot of the differentially accumulated metabolites under different treatments: **(A)** Cd treatment vs. control, **(B)** La treatment vs. control, **(C)** Cd + La treatment vs. Cd treatment. Each origin in the volcano plot represents a metabolite: the red origin denotes up-regulated metabolites, and the blue bar refers to down-regulated metabolites. The abscissa represents the logarithmic fold change of metabolites in two groups. Ordinate represents the significance level of different metabolites.

**Figure 6:**
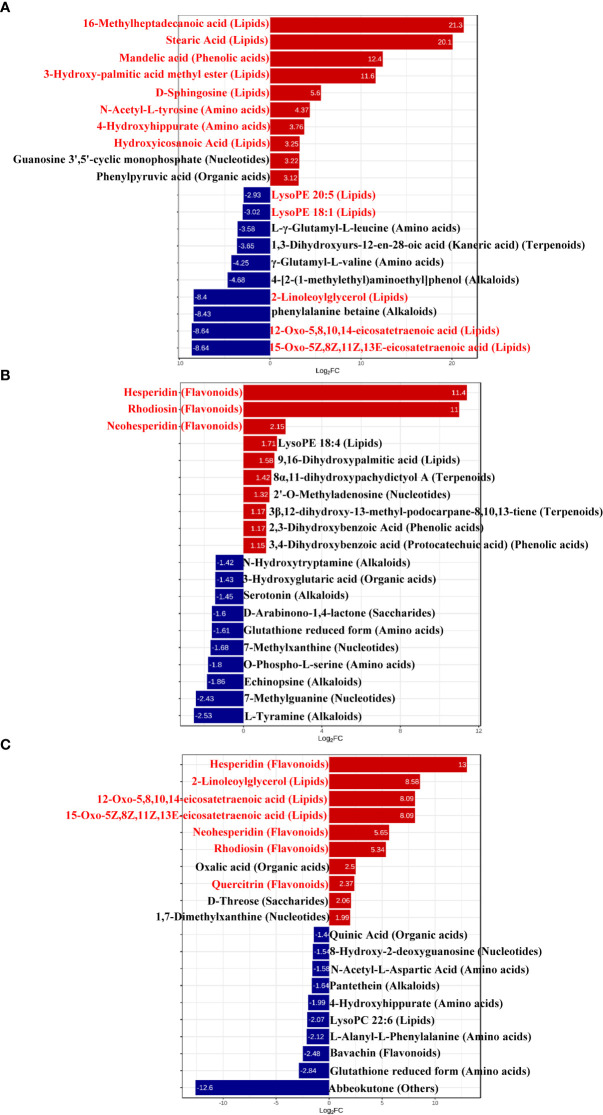
Bar chart of top differentially accumulated metabolites under different treatments: **(A)** Cd treatment vs. control, **(B)** La treatment vs. control, **(C)** Cd + La treatment vs. Cd treatment. The ordinate is the name of the DAMs; the abscissa is the Log_2_FC (fold change), representing the logarithmic fold change of metabolites in two groups. The red bar denotes the metabolites up-regulated, and the blue bar signifies the metabolites down-regulated. Red letters stand for metabolites for subsequent analysis.

**Figure 7:**
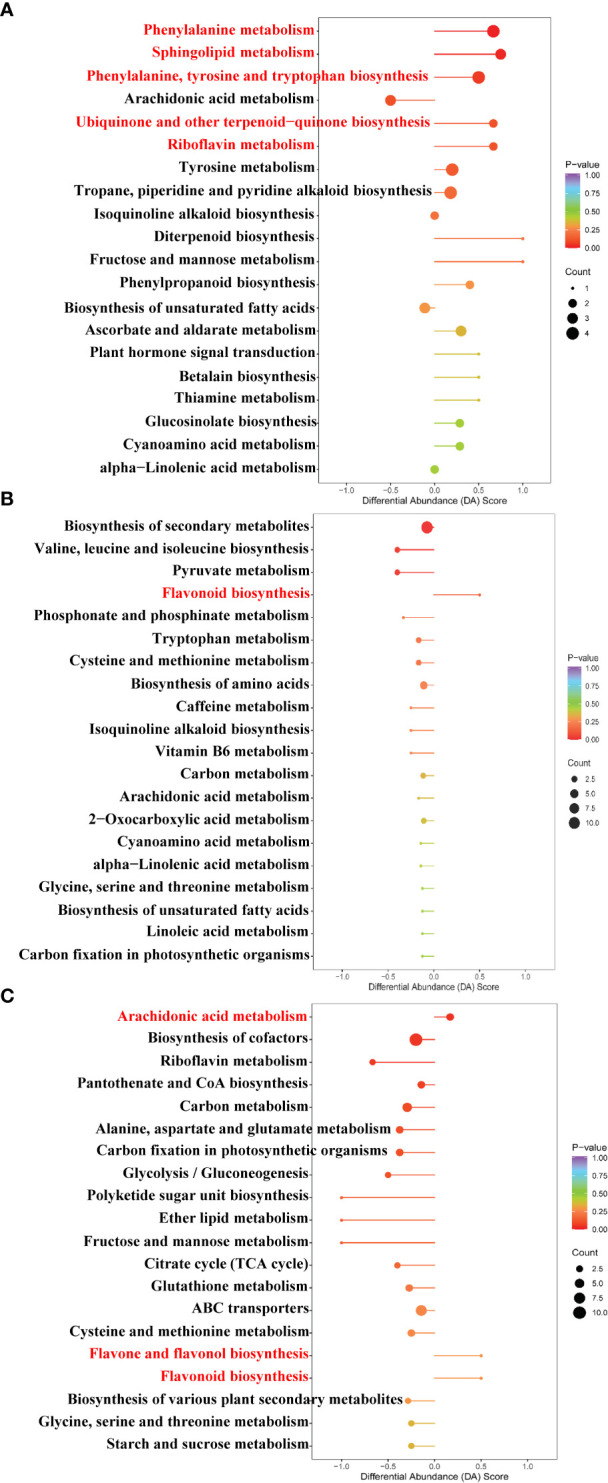
Bubble chart of the top enriched metabolic pathway under different treatments: **(A)** Cd treatment vs. control, **(B)** La treatment vs. control, **(C)** Cd + La treatment vs. Cd treatment. The ordinate is the name of the metabolic pathway; the abscissa is the DA Score (the differential abundance score). DA Score extending to the right indicates that the pathway is up-regulated, otherwise indicates that the pathway is down-regulated. A larger dot indicates that more metabolites are enriched in that pathway. The colour from purple to red indicates that the p-value decreases. The red letters denote metabolic pathways for subsequent analysis.

To better explore the DAMs in samples where La can alleviate the Cd toxicity of accumulated metabolites, the DAMs of *G. bailiniae* treated with the control, Cd, La, and Cd + La groups were analysed using the UPLC-MS technique. The multiple reaction monitoring (MRM) results and total ion chromatography (TIC) are shown in **Figures S2** and **S3**, respectively. The unsupervised principal component analysis (PCA) and supervised orthogonal partial least squares discriminant analysis (OPLS-DA) outcomes indicate that DAMs of *G. bailiniae* could be differentiated by enforcing different treatments for seven days (**Figures S4**, **S5**). The PCA and OPLS-DA demonstrated that DAMs of G. bailiniae within the same treatment were assembled and gathered together because of their slight differences and close distances. In addition, a distinct separation occurred during the application of the different treatments. More specifically, it can be argued from the extracted results that different treatments resulted in changes in the DAMs contents and the type of DAMs. The methods of 7-fold cross-validation and response permutation testing (RPT) were used to test the employed model’s quality and indicated no overfitting in the measurement model. The R^2^Y and Q^2^ parameters exhibited better stability and predictability. The volcano plot was also used to visualize the DAMs under positive and negative ion modes. As shown in **Figure 5**, the red origin represents the significantly up-regulated DAMs in the experimental group, the blue origin refers to the significantly down-regulated DAMs, and the gray point denotes the non-significant DAMs. By using VIP≥ 1 and *p* values ≤0.05 screening, a total of 321 DAMs were isolated in the Cd treatment compared with the control, in which 168 DAMs were up-regulated (red dots), and 153 DAMs were down-regulated (blue dots), respectively (**Figure 5A**). Two-hundred-and-twelve differential metabolites were isolated in the La treatment compared with the control, in which 111 DAMs were up-regulated (red dots), while 101 of the differential metabolites were down-regulated (blue dots), respectively (**Figure 5B**). Additionally, 266 of the DAMs were isolated in the Cd + La treatment compared with the application of the Cd treatment, with 101 of the DAM up-regulated (red dots), and 165 of the DAMs were down-regulated (blue dots), respectively (**Figure 5C**). The top 10 up- and down-regulation DAMs are shown in **Figures 7A**–**7C**, and their detailed information is listed in **Tables S1** to **S3**. The majority of the top 10 up- and down-regulated DAMs of *G. bailiniae* are lipids (16-Methylheptadecanoic acid, Stearic Acid, 3-Hydroxy-palmitic acid methyl ester, D-Sphingosine, Hydroxyicosanoic Acid, 15-Oxo-5Z,8Z,11Z,13E-eicosatetraenoic acid, 12-Oxo-5,8,10,14-eicosatetraenoic acid, 2-Linoleoylglycerol, LysoPE 18:1 and LysoPE 20:5) and amino acids (N-Acetyl-L-tyrosine, 4-Hydroxyhippurate, γ-Glutamyl-L-valine, and L-γ-Glutamyl-L-leucine) in the Cd treatment compared to the control (**Figure 6A**). Notably, 1 phenolic acids (Mandelic acid) is significantly up-regulated as a result of the Cd treatment. Flavonoids (Hesperidin, Rhodiosin, and Neohesperidin) are also significantly up-regulated in *G. bailiniae* following La treatment compared to the control group (**Figure 6B**). Concerning the Cd + La treatment, flavonoids (Hesperidin, Neohesperidin, Rhodiosin, and Quercitrin) and lipids (2-Linoleoylglycerol, 12-Oxo-5,8,10,14-eicosatetraenoic acid, and 15-Oxo-5Z,8Z,11Z,13E-eicosatetraenoic acid) are the dominant DAMs in the top 10 up-regulated DAMs of *G. bailiniae* compared to the control samples (**Figure 6C**).

### Impact of Cd, La, and their combination on *G. bailiniae* metabolic pathway

The pathway enrichment analysis of DAMs was carried out according to the Kyoto Encyclopedia of Genes and Genomes (KEGG) database. As seen in **Figure 7A**, the top five up-regulated pathways of the Cd treatment compared to the control were involved in phenylalanine metabolism, sphingolipid metabolism, phenylalanine, tyrosine, and tryptophan biosynthesis, ubiquinone, and other terpenoid–quinone biosyntheses and riboflavin metabolism. As can be observed from **Figure 7B**, the up-regulated pathway of the La treatment compared to the control was involved in the flavonoid biosynthesis. Moreover, as can be detected from **Figure 7C**, the up-regulated pathway enrichment of the Cd + La treatment compared to the Cd treatment was involved in arachidonic acid metabolism, flavone and flavonol biosynthesis, and flavonoid biosynthesis.

## Discussion

Cadmium is considered a primary marine pollutant that accumulates in macroalgae and threatens human health worldwide through the food chain (Costa et al., 2020). For humans, Alzheimer, Parkinson, and Itai-itai are infamous Cd-induced diseases. Osteoporosis, renal dysfunction, and bladder cancer have also been reported to be induced by Cd (Akesson et al., 2008; Feki-Tounsi and Hamza-Chafai, 2014; Meira and Dias, 2017). Cd is also known to inhibit macroalgae’s typical growth and photosynthetic function. In the work of Xia et al. (2004), 100 μM Cd significantly suppressed the growth, photosynthetic O_2_ evolution, and pigment content of *Gracilaria lemaneiformis*. Notably, Zhu et al. (2011; 2017) reported that 4 – 10 mg·L^−1^ of Cd could markedly reduce the growth and the photosynthetic activity of *Hizikia fusiformis* and *Porphyra haitanensis*. It should be highlighted that Cd has no known metabolic function in algae and cannot contribute to the ˙OH formation in the Fenton reaction (Halliwell and Gutteridge, 2015). Nevertheless, Cd is taken up by algae, resulting in disturbances in photosynthesis and a reduction in RGR (Collén et al., 2003; Zhu et al., 2011). Additionally, several biological toxicities of La on different microalgae and macroalgae have been reported, but the information is sparse and incomplete (Liu et al., 1986; Jin et al., 2009; Sun et al., 2019; Figueiredo et al., 2022). Cadmium and La are widely recognized as biphasic hormones in higher terrestrial plants, stimulating plant growth at low doses and inhibiting it at high doses, with differences between the various species (Pagano et al., 2015; Agathokleous et al., 2018; Carvalho et al., 2020; Noor et al., 2022). In the present work, 5 mg·L^−1^ of Cd treatment markedly suppresses the growth and photosynthetic efficiency of *G. bailiniae*. Compared to the control group, 8 mg·L^−1^ La treatment resulted in increased growth and photosynthetic efficiency of *G. bailiniae*. Further, the presence of La could effectively decrease the Cd accumulation and alleviate the adverse impact of Cd on the growth and the photosynthetic efficiency of *G. bailiniae*, which was similar to previous reports on alleviation of La to the higher terrestrial plants under Cd stress (He et al., 2005; Wang et al., 2012). Sulphated compounds were also present in the cell walls of red algae, whereas an increased thickness of the cell wall is a chelation strategy to effectively protect the algae (Dos Santos et al., 2014). Once the chelation of metals by the cell walls is insufficient, the algal cellular interior will show heterogeneities, such as an increase in plastoglobuli, starch grains, vacuolization, and disrupted organelle structures (Bouzon et al., 2012; Dos Santos et al., 2013; Dos Santos et al., 2014). The accumulation of starch grains is a detoxification mechanism to resist stresses and maintain the expected, normal growth of algae (Li et al., 2021). Moreover, increased starch grains could be associated with morphological changes, such as a decrease in thylakoids of *Hypnea musciformis* cells exposed to 50 μM cadmium. Notably, cells treated with cadmium at 200 μM and 300 μM revealed no starch grains in either cortical or subcortical cells (Bouzon et al., 2012). As seen from the provided TEM analysis, applying 5 mg·L^−1^ of Cd increased the plastoglobuli number and the cortical cell wall thickness, disrupted the chloroplast and mitochondria, and decreased starch grains number. Apart from the cortical cell appearing completely vacuolated, the implementation of 8 mg·L^−1^ of La treatment showed negligible impact on the ultrastructure of the cortical cell. Except for starch grains, the cellular and organelle structure of the cortical cell in the Cd and La combination treatment was more intact and well-shaped than that in the Cd treatment. Starch grains were also significantly increased and swollen compared to the control, with both outcomes inferring that low concentrations of Cd treatment could promote starch grain synthesis.

Chlorophylls are the fundamental pigments in algae’s light absorption and photochemistry function (Eullaffroy and Vernet, 2003). Carotenoids, accessory pigments in algae, serve as light-harvesting pigments during photosynthesis and protective molecules against photo-oxidative damage (Bryant, 1996). Both PE and PC, fluorescent proteins in the auxiliary photosynthetic complexes of red macroalgae, can also capture light energy (Dagnino-Leone et al., 2022). Chl a, carotenoid, PE, and PC are the primary photosynthetic pigments of red macroalgae and are critical targets for metals. For that reason, they are frequently used to assess metal stress (Xia et al., 2004; Zhu et al., 2011). Greger and Ögren (1991) found that the Cd treatment could cause magnesium (Mg) and iron deficiency (Fe) during the process of chlorophyll biosynthesis, resulting in the reduction of the chlorophyll content. Low concentrations of REEs were found to positively impact the chlorophyll content of algae (Shen et al., 2018). Collén et al. (2003) and Saleh (2015) reported that the accumulation of carotenoid pigments under heavy metals stress could be considered a mechanism developed by algae involved in its stress tolerance. In contrast to chl a and carotenoids, PE and PC are more sensitive to Cd stress (Zhu et al., 2017). This phenomenon has also been reported in *G. lemaneiformis* (Xia et al., 2004). Increasing concentrations of PE and PC may also indicate an effective protective mechanism that can accelerate the photosynthesis process. The decrease in PE and PC contents is possibly due to the phycobilisomes being destroyed by implementing a relatively high Cd treatment (Xia et al., 2004). In the present work, 5 mg·L^−1^ Cd treatment markedly suppressed the content of chl a, PE, and PC, but carotenoid content increased. Compared to the control, applying the 8 mg·L^−1^ La treatment increased pigment content (chl a, carotenoid, PE, and PC). The combined Cd and La treatment not only effectively alleviates the decrease in chl a, PE, and PC but also inhibits the accumulation of carotenoids compared to the Cd treatment.

Reactive oxygen species (ROS) are considered cellular aerobic metabolism products of organisms (Regoli and Giuliani, 2014). Various environmental stresses, including pollutants exposure, can lead to excessive production of ROS, causing peroxidation damage (Bojarski et al., 2020). To avoid excessive cellular damage, algae activate defense mechanisms, namely enhancing the enzymatic (the activity of the antioxidant enzymes system) and nonenzymatic reactions, aiming to eliminate the excess ROS (Zhu et al., 2017). SOD is a vital enzyme in the first line of defense against ROS. More specifically, it can convert the superoxide radicals to less toxic agents, producing H_2_O_2_ (Gill et al., 2015). CAT and POD are essential enzymes that scavenge excess H_2_O_2_ by catalyzing it to produce H_2_O and O_2_ for ROS removal (Garg and Manchanda, 2009). ROS overproduction and inefficient elimination by antioxidant mechanisms may induce the peroxidation of membrane lipids (Trapasso et al., 2021). Among the peroxidised molecules generated during the lipid peroxidation process, malondialdehyde (MDA) is considered one of the most abundant and probably the most commonly utilized as an oxidative stress marker (Regoli et al., 2011; Regoli and Giuliani, 2014). As was demonstrated in the present work’s antioxidant enzyme activity and lipid peroxidation level analysis, Cd exposure caused an increased antioxidant enzyme activity and lipid peroxidation. Compared to the control group, the application of 8 mg·L^−1^ La treatment slightly increased the antioxidant enzyme activity (SOD and POD) and negatively impacted lipid peroxidation. The combination of the Cd and La treatment effectively alleviated the increased antioxidant enzyme activity and lipid peroxidation levels compared to the Cd treatment.

Membrane lipids are often modified qualitatively and quantitatively under metal stress, including the accumulation of unsaturated fatty acids and changes in the total and relative abundance of the various cell phospholipids and plasma membranes (Devi and Prasad, 1999). The accumulation of some lipids might be an immediate defense to alleviate heavy metal stress and maintain normal growth rates (Yu et al., 2012; Leong and Chang, 2020). In the present work, the significant up-regulated five lipids (16-Methylheptadecanoic acid, Stearic Acid, 3-Hydroxy-palmitic acid methyl ester, D-Sphingosine, and Hydroxyicosanoic Acid) of *G. bailiniae* in the Cd treatment groups might contribute to enhanced antioxidant ability, membrane defense, and cellular programmed regulation to alleviate the Cd stress (Hernandez and Cooke, 1997; Howlett and Avery, 1997; Devi and Prasad, 1999; Khan et al., 2013; Narayanan and Park, 2013; Michaelson et al., 2016; Dwivedi et al., 2017). However, such defense types were insufficient, resulting in another five lipids (15-Oxo-5Z,8Z,11Z,13E-eicosatetraenoic acid, 12-Oxo-5,8,10,14-eicosatetraenoic acid, 2-Linoleoylglycerol, LysoPE 18:1, and LysoPE 20:5) being significantly down-regulated. Such down-regulation may be attributed to the destruction of organelle membranes, plasma membranes, and redox homeostasis, resulting in physiological disorder (Devi and Prasad, 1999; Zhang et al., 2019; Tan et al., 2022). Additionally, the up-regulation of phenolic acids (Mandelic acid) and amino acids (N-acetyl-L-tyrosine and 4-Hydroxyhippurate) might contribute to both chelation and antioxidation of *G. bailiniae* in the Cd treatment group (Tomás-Barberán and Espín, 2001; Michalak, 2006; Gülçin, 2007; Nieman et al., 2015; Yang and Shen, 2020; Khan et al., 2021). Furthermore, DAMs up-regulated pathways were mainly concentrated in phenylalanine metabolism, sphingolipid metabolism, phenylalanine, tyrosine, and tryptophan biosynthesis, ubiquinone, and another terpenoid−quinone biosynthesis, as well as riboflavin metabolism. These up-regulated pathways of *G. bailiniae* are mainly involved in antioxidation, membrane defense, metal chelation, and cellular-programmed regulation in the Cd treatment group (Lu et al., 2004; Vogt, 2010; Fan et al., 2014; Deng and Lu, 2017; Li et al., 2018; Guo et al., 2019; Cheng et al., 2021; Wang et al., 2022). This data was further validated by the results of significant DAMs in the Cd treatment compared to the control. Flavonoids, low molecular weight polyphenolic secondary metabolic compounds, protect algae and plants against various biotic and abiotic stresses, exhibiting a diverse spectrum of biological functions which may also play an important role in the interaction between the algae (or plant) and their environments (Samanta et al., 2011; Fernando et al., 2022). As a flavone compound, Hesperidin can be easily synthesized in algae and plants under environmental stress, such as heavy metal stress and temperature change, as ROS scavengers (Jeon et al., 2014; Xie et al., 2020). Moreover, hesperidin could protect photosynthesis reactions in mesophyll cells by inhibiting the accumulation of metal ions and simultaneously maintaining the activation of crucial enzymes, which might trigger the renewal of the impaired chlorophyll molecules and improved RGR levels (Arikan et al., 2022). In addition, hesperidin is regarded as the metabolic engineering precursor of neohesperidin (Ververidis et al., 2007). Neohesperidin is a flavanone biosynthesized in many algae and plants as a potent antioxidant (Tang et al., 2019; Tahir et al., 2022). Rhodiosin, an antioxidant flavonol glycoside from Rhodiola rosea, could significantly eliminate hydroxyl and ${\text{O}}_{2}^{-}$ and decrease lipid peroxidation (Kwon et al., 2009). In the current study, the most significant up-regulated DAMs in *G. bailiniae* are three flavonoids (Hesperidin, Rhodiosin, and Neohesperidin) in the La treatment. These DAMs mainly displayed inhibition of the Cd accumulation, renewal of the impaired chlorophyll molecule, maintenance of the crucial enzyme activity, enhancement of antioxidant ability, and improvement of growth. Furthermore, DAMs up-regulated pathways were mainly focused on flavonoid biosynthesis, supporting the results of the significant up-regulated DAMs in the La treatment. Quercitrin is also considered an antioxidant flavonol in algae and plants experiencing metal stress (Skórzy ska-Polit et al., 2004; El-Tablawy et al., 2020). Finally, eicosatetraenoic acid is considered an evolutionarily conserved defense signalling molecule that modulates algae and plant stress signalling networks (Shanab et al., 2018; Tan et al., 2022). In the present work, four flavonoids (Hesperidin, Neohesperidin, Rhodiosin, and Quercitrin) and three lipids (2-Linoleoylglycerol, 12-Oxo-5,8,10,14-eicosatetraenoic acid, and 15-Oxo-5Z,8Z,11Z,13E-eicosatetraenoic acid) were significantly up-regulated in the Cd + La treatment group, compared to the Cd treatment group, which might be attributable to the La mediated biosynthesis of flavonoids and lipids. These DAMs could inhibit Cd accumulation, modulate the plant stress signalling networks, renew the impaired chlorophyll molecules, maintain crucial enzyme activities, enhance antioxidant efficiency, stabilize the redox homeostasis and improve overall growth. Furthermore, DAMs up-regulated pathways were mainly concentrated in arachidonic acid metabolism, flavone and flavonol biosynthesis, and flavonoid biosynthesis, which validated the results of the significant up-regulated DAMs in Cd + La treatment group, compared to the Cd treatment samples.

## Conclusions

In this work, *G. bailiniae* could enhance self-antioxidant ability, membrane defense, and cellular-programmed regulation to compensate for the Cd stress. The presence of La mediates the biosynthesis of flavonoids and lipids, significantly inhibiting the Cd accumulation, modulating algal stress signalling networks, renewing the impaired chlorophyll molecules, maintaining the crucial enzyme activities, enhancing the antioxidant capabilities, stabilising redox homeostasis and improving overall growth. Consequently, La should be considered a Cd detoxicant, which enhances the stress tolerance and adaptability of *G. bailiniae* in aquatic environments, promoting a broader application of *G. bailiniae* in wastewater treatment and eco-environmental modification.

## Data availability statement

The original contributions presented in the study are included in the article/**Supplementary Material**, further inquiries can be directed to the corresponding author/s.

## Author contributions

Data curation, BH formal analysis, EX and JC. Funding acquisition, EX and JC. Investigation, BH, JC, YR, CC, FL, YZ, ZL, and EX. Methodology, BH and JC. Project administration, EX and JC. Resources, EX and JC. Writing—original draft, BH. Writing—review & editing, BH and JC. All authors contributed to the article and approved the submitted version.

## Funding

This study was sponsored by the National Key Research and Development Program of China (2020YFD0901101), PhD Start-up Foundation of Guangdong Ocean University (R19049), Science and Technology planning project of Guangdong (2017A030303078), and Undergraduate Start-up Foundation of Guangdong Ocean University (580520135 and 570119011).

## Acknowledgments

We thank Wuhan Metware Biotechnology Co., Ltd. for providing the data analysis tools for this study.

## Conflict of interest

The authors declare that they have no known competing financial interests or personal relationships that could have appeared to influence the work reported in this paper.

## Publisher’s note

All claims expressed in this article are solely those of the authors and do not necessarily represent those of their affiliated organizations, or those of the publisher, the editors and the reviewers. Any product that may be evaluated in this article, or claim that may be made by its manufacturer, is not guaranteed or endorsed by the publisher.
