# Supplementary material for: Mechanism of macroalgae Gracilaria bailiniae responding to cadmium and lanthanum
Source: Front Plant Sci. 2022 Dec 1;13:1076526. doi: 10.3389/fpls.2022.1076526 (PMC9756850; doi:10.3389/fpls.2022.1076526)
Supplement: Supplementary file 1 [file DataSheet_1.pdf]

Supplementary Material

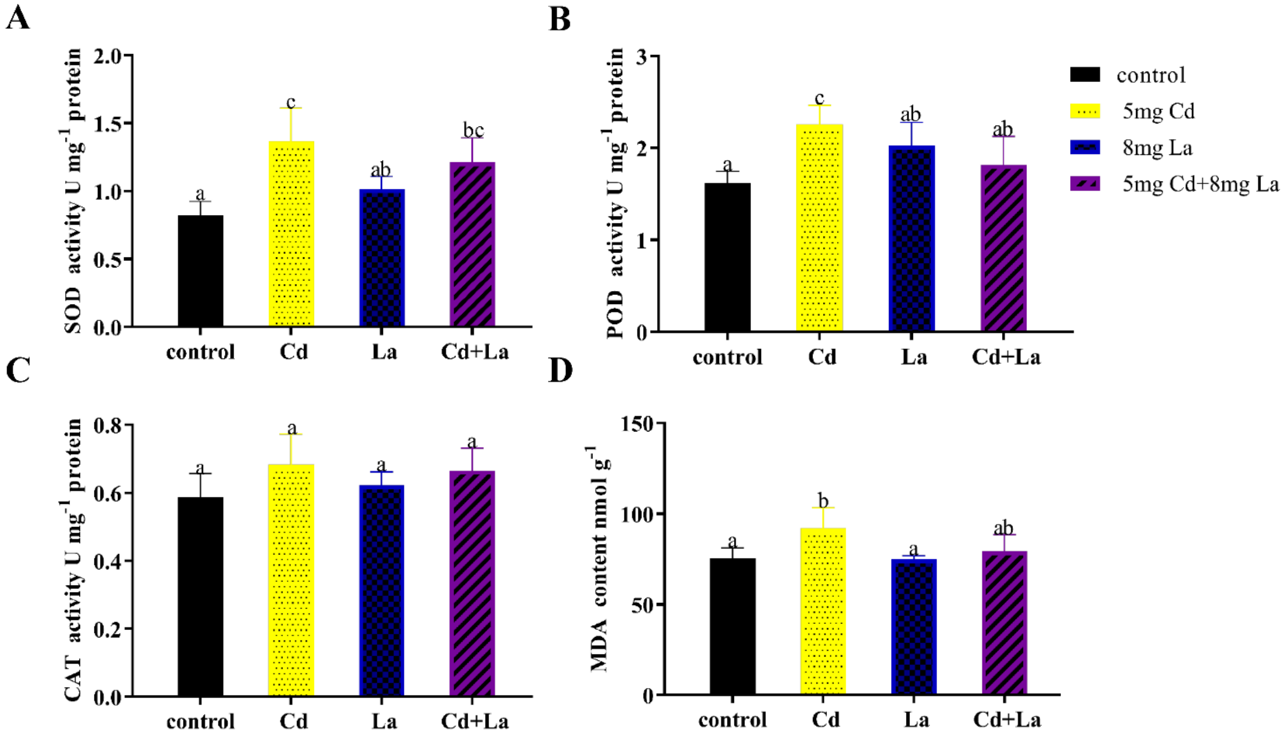

Fig. S1. Antioxidant enzyme activity and lipid peroxidation level of *G. bailiniae* exposed to Cd, La, and their combination: (A) SOD, (B) POD, (C) CAT, (D) MDA. The data are presented as the mean ± standard deviation (n = 3). The lowercase letters indicate significant differences (ANOVA/Duncan ANOVA, $P < 0.05$).

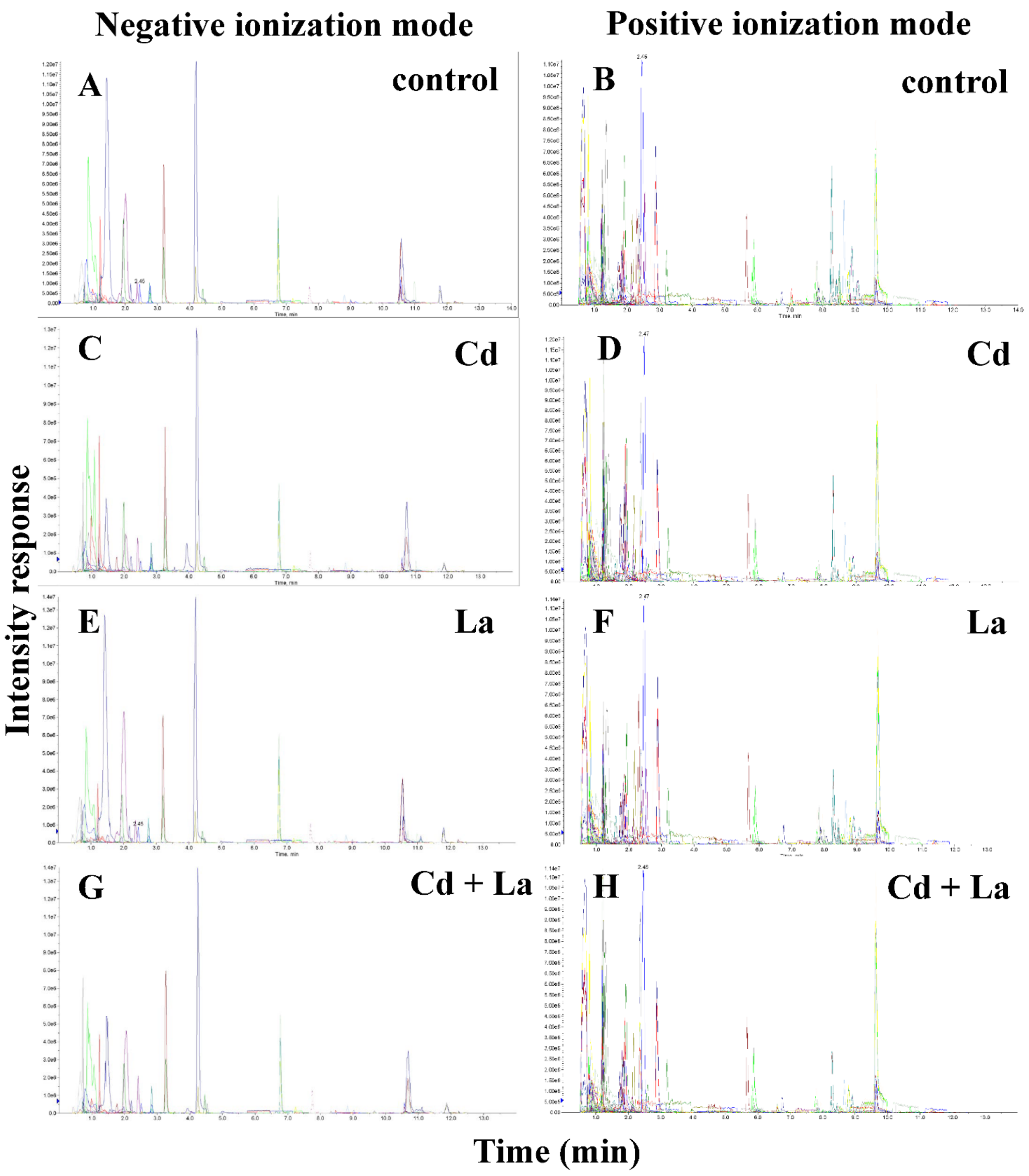

Fig. S2. Multiple reaction monitoring (MRM) of accumulated metabolites under different treatment in negative and positive ionization mode. The ordinate and abscissa represent the intensity response and time of accumulated metabolites, respectively.

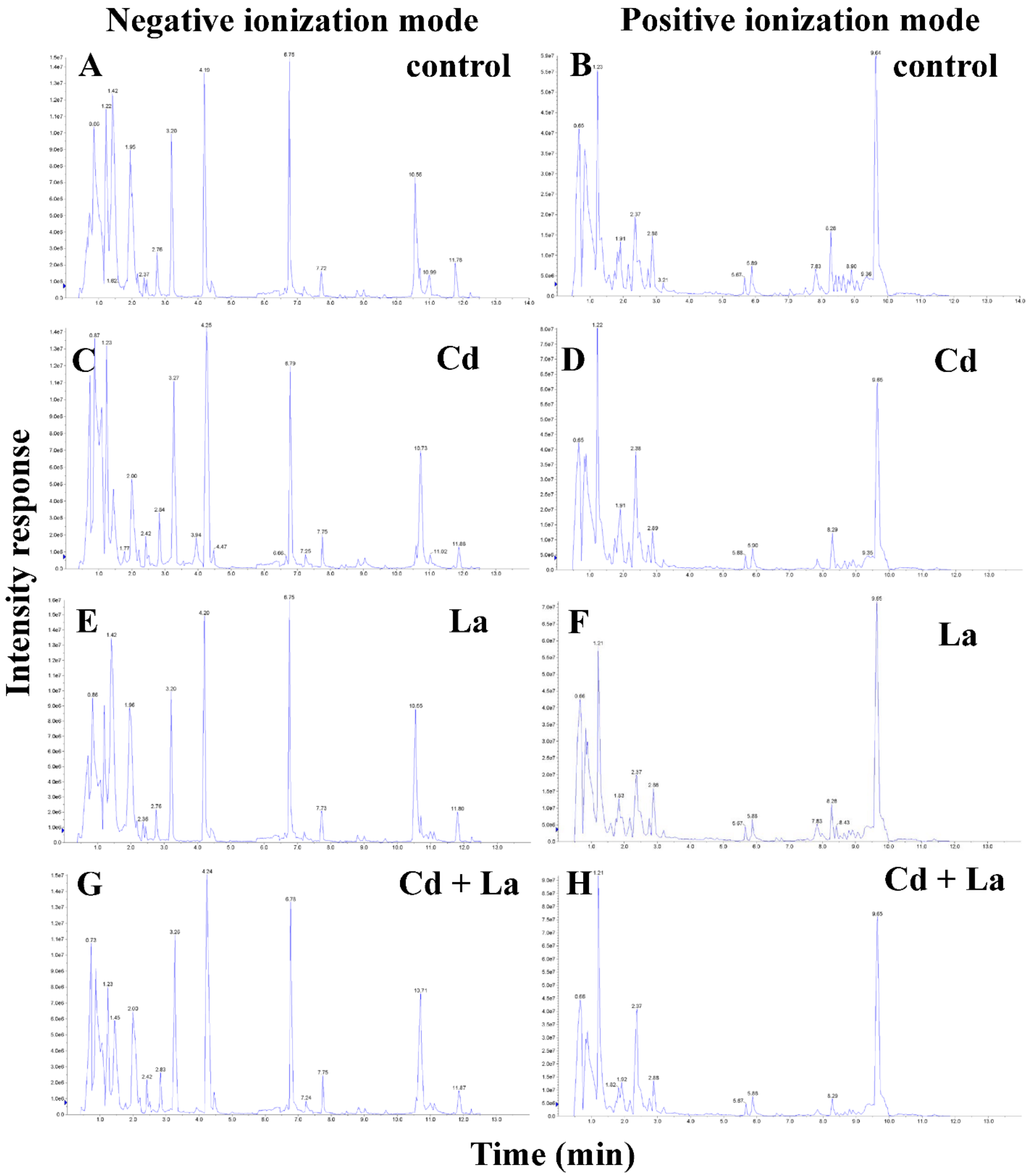

Fig. S3. Total ion chromatography (TIC) of accumulated metabolites under different treatment in negative and positive ionization mode. The ordinate and abscissa represent the intensity response and time of accumulated metabolites, respectively.

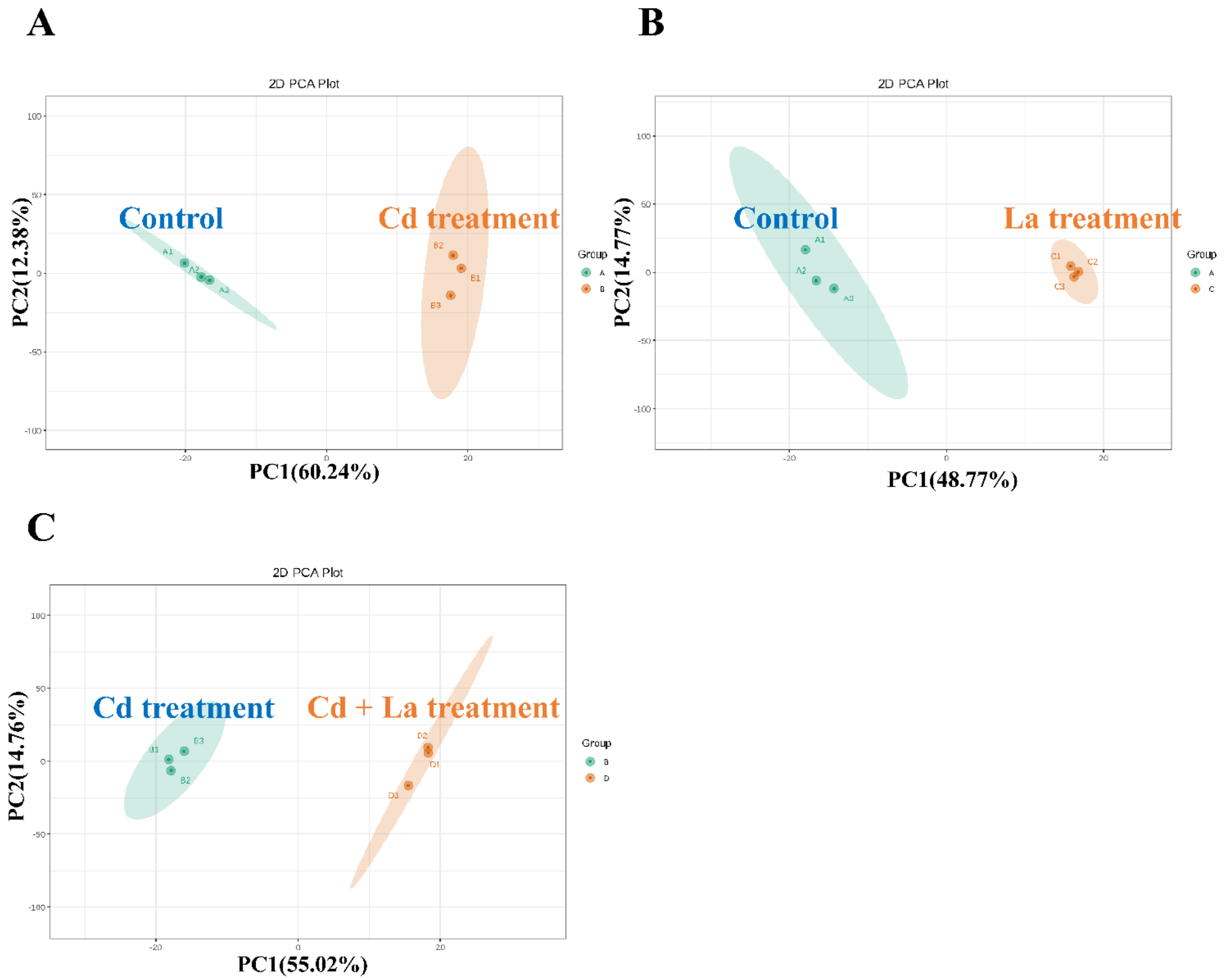

Fig. S4. principal component analysis (PCA) of the differentially accumulated metabolites under different treatment: (A) Cd treatment vs control, (B) La treatment vs control, (C) Cd + La treatment vs Cd treatment. PC1 and PC2 represent the first and second principal component, respectively. The percentage indicates the effect rate of principal component to the dataset.

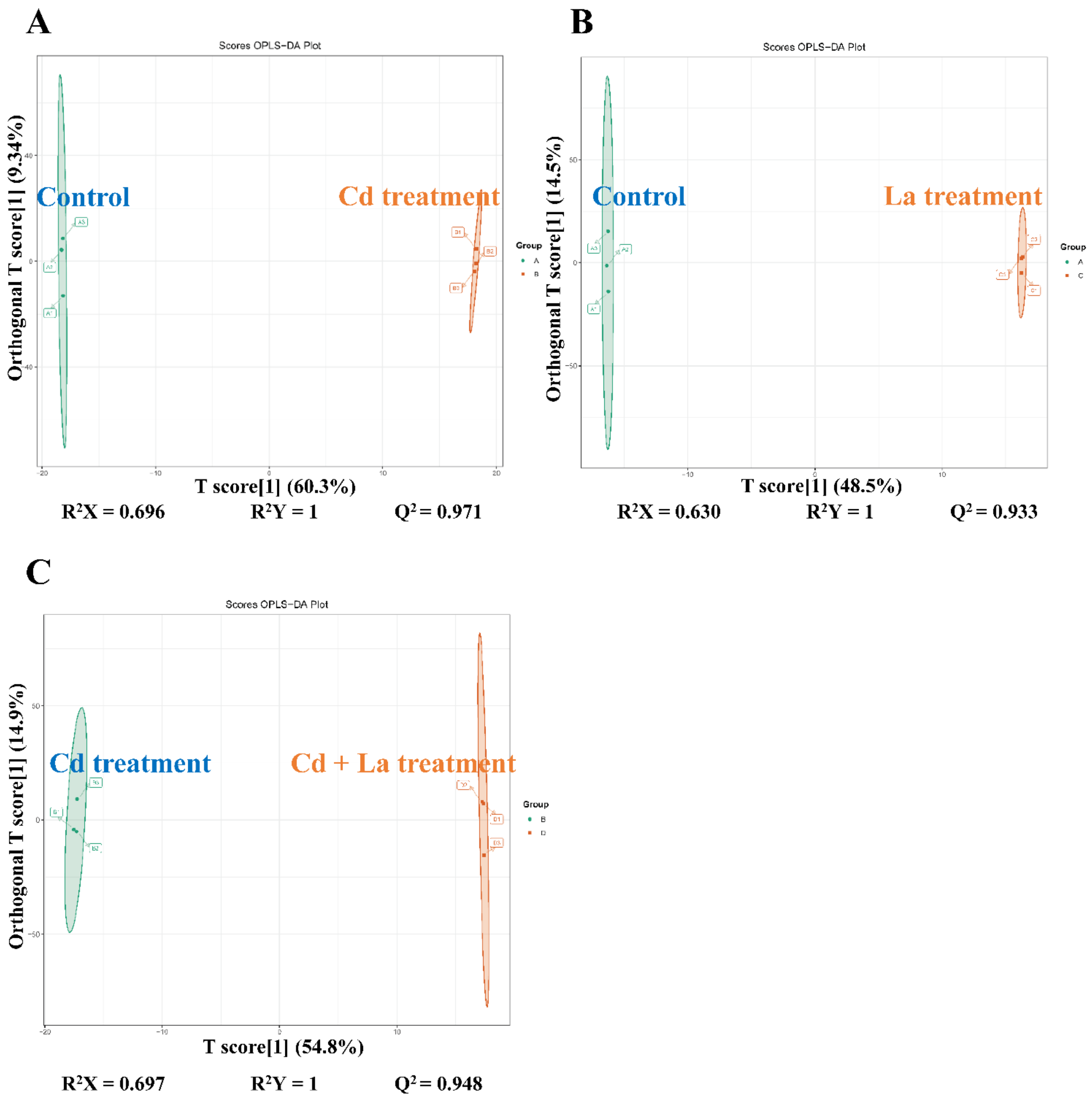

Fig. S5. Orthogonal partial least squares discriminant analysis (OPLS-DA) of the differentially accumulated metabolites under different treatment: (A) Cd treatment vs control, (B) La treatment vs control, (C) Cd + La treatment vs Cd treatment. Orthogonal T score[1] and T score[1] represent the orthogonal and predict principal component, respectively. The percentage indicates the effect rate of principal component to the dataset. The prediction parameters of OPLS-DA model are R^2X, R^2Y and Q^2. R^2X and R^2Y represent the effect rate of OPLS-DA model to the X and Y matrices, respectively. Q^2 represents the predictive ability of OPLS-DA model, Q^2 >0.5 can be considered an available model, and Q2 >0.9 is considered as an excellent model.

Table S1 The top 10 up- and down-regulation DAMs of Cd treatment vs control

Compounds	Formula	Class	CAS	VIP	Log2FC	Type
16-Methylheptadecanoic acid	$C_{18}H_{36}O_2$	Lipids	2724-58-5	1.29	21.3	up
Stearic Acid	$C_{18}H_{36}O_2$	Lipids	57-11-4	1.29	20.1	up
Mandelic acid	$C_8H_8O_3$	Phenolic acids	90-64-2	1.29	12.4	up
3-Hydroxy-palmitic acid methyl ester	$C_{17}H_{34}O_3$	Lipids	51883-36-4	1.29	11.6	up
D-Sphingosine	$C_{18}H_{37}NO_2$	Lipids	123-78-4	1.28	5.60	up
N-Acetyl-L-tyrosine	$C_{11}H_{13}NO_4$	Amino acids	537-55-3	1.29	4.37	up
4-Hydroxyhippurate	$C_9H_9NO_4$	Amino acids	2482-25-9	1.26	3.76	up
Hydroxyicosanoic Acid	$C_{20}H_{40}O_3$	Lipids	/	1.28	3.25	up
Guanosine 3',5'-cyclic monophosphate	$C_{10}H_{12}N_5O_7P$	Nucleotides	7665-99-8	1.28	3.22	up
Phenylpyruvic acid	$C_9H_8O_3$	Organic acids	156-06-9	1.29	3.12	up
LysoPE 20:5	$C_{25}H_{42}NO_7P$	Lipids	/	1.26	-2.93	down
LysoPE 18:1	$C_{23}H_{46}NO_7P$	Lipids	89576-29-4	1.27	-3.02	down
L-γ-Glutamyl-L-leucine	$C_{11}H_{20}N_2O_5$	Amino acids	2566-39-4	1.28	-3.58	down
Kaneric acid	$C_{30}H_{48}O_4$	Terpenoids	107693-87-8	1.27	-3.65	down
γ-Glutamyl-L-valine	$C_{10}H_{18}N_2O_5$	Amino acids	/	1.29	-4.25	down
4-[2-(1-methylethyl)aminoethyl]phenol	$C_{11}H_{17}NO$	Alkaloids	/	1.29	-4.68	down
2-Linoleoylglycerol*	$C_{21}H_{38}O_4$	Lipids	3443-82-1	1.29	-8.40	down
phenylalanine betaine	$C_{12}H_{17}NO_2$	Alkaloids	56755-22-7	1.29	-8.43	down
12-Oxo-5,8,10,14-eicosatetraenoic acid	$C_{20}H_{30}O_3$	Lipids	108437-64-5	1.29	-8.64	down
15-Oxo-5Z,8Z,11Z,13E-eicosatetraenoic acid	$C_{20}H_{30}O_3$	Lipids	81416-72-0	1.29	-8.64	down

VIP denotes the weight value of the variable from the OPLS-DA model. Generally, the metabolites of VIP > 1 are considered as DAMs. log_2(FC): the ratio of the average expression of metabolites in the two groups of samples. Positive value means up-regulation; negative value means down-regulation FC fold change. / denotes none corresponding number.

Table S2 The top 10 up- and down-regulation DAMs of La treatment vs control

Compounds	Formula	Class	CAS	VIP	Log2FC	Type
Hesperidin	$C_{28}H_{34}O_{15}$	Flavonoids	520-26-3	1.43	11.4	up
Rhodiosin	$C_{27}H_{30}O_{16}$	Flavonoids	86831-54-1	1.44	11.0	up
Neohesperidin	$C_{28}H_{34}O_{15}$	Flavonoids	13241-33-3	1.18	2.15	up
LysoPE 18:4	$C_{23}H_{40}NO_7P$	Lipids	/	1.40	1.71	up
9,16-Dihydroxypalmitic acid	$C_{16}H_{32}O_4$	Lipids	38076-49-2	1.40	1.58	up
8α,11-dihydroxypachydictyol A	$C_{20}H_{32}O_3$	Terpenoids	/	1.43	1.42	up
2'-O-Methyladenosine	$C_{11}H_{15}N_5O_4$	Nucleotides	2140-79-6	1.40	1.32	up
3β,12-dihydroxy-13-methyl-podocarpane-8,10,13-tiene	$C_{18}H_{26}O_2$	Terpenoids	/	1.43	1.17	up
2,3-Dihydroxybenzoic Acid	$C_7H_6O_4$	Phenolic acids	303-38-8	1.40	1.17	up
Protocatechuic acid	$C_7H_6O_4$	Phenolic acids	99-50-3	1.37	1.15	up
N-Hydroxytryptamine	$C_{10}H_{12}N_2O$	Alkaloids	/	1.43	-1.42	down
3-Hydroxyglutaric acid	$C_5H_8O_5$	Organic acids	638-18-6	1.38	-1.43	down
Serotonin	$C_{10}H_{12}N_2O$	Alkaloids	50-67-9	1.41	-1.45	down
D-Arabinono-1,4-lactone	$C_5H_8O_5$	Saccharides	2782-09-4	1.42	-1.60	down
Glutathione reduced form	$C_{10}H_{17}N_3O_6S$	Amino acids	70-18-8	1.41	-1.61	down
7-Methylxanthine	$C_6H_6N_4O_2$	Nucleotides	552-62-5	1.40	-1.68	down
O-Phospho-L-serine	$C_3H_8NO_6P$	Amino acids	407-41-0	1.29	-1.80	down
Echinopsine	$C_{10}H_9NO$	Alkaloids	83-54-5	1.34	-1.86	down
7-Methylguanine	$C_6H_7N_5O$	Nucleotides	578-76-7	1.44	-2.43	down
L-Tyramine	$C_8H_{11}NO$	Alkaloids	51-67-2	1.42	-2.53	down

VIP denotes the weight value of the variable from the OPLS-DA model. Generally, the metabolites of VIP > 1 are considered as DAMs. log_2(FC): the ratio of the average expression of metabolites in the two groups of samples. Positive value means up-regulation; negative value means down-regulation FC fold change. / denotes none corresponding number.

Table S3 The top 10 up- and down-regulation DAMs of Cd + La treatment vs Cd treatment

Compounds	Formula	Class	CAS	VIP	Log2FC	Type
Hesperidin	$C_{28}H_{34}O_{15}$	Flavonoids	520-26-3	1.35	13.0	up
2-Linoleoylglycerol	$C_{21}H_{38}O_4$	Lipids	3443-82-1	1.35	8.58	up
12-Oxo-5,8,10,14-eicosatetraenoic acid	$C_{20}H_{30}O_3$	Lipids	108437-64-5	1.35	8.09	up
15-Oxo-5Z,8Z,11Z,13E-eicosatetraenoic acid	$C_{20}H_{30}O_3$	Lipids	81416-72-0	1.35	8.09	up
Neohesperidin	$C_{28}H_{34}O_{15}$	Flavonoids	13241-33-3	1.34	5.65	up
Rhodiosin	$C_{27}H_{30}O_{16}$	Flavonoids	86831-54-1	1.08	5.34	up
Oxalic acid	$C_2H_2O_4$	Organic acids	144-62-7	1.28	2.50	up
Quercitrin	$C_{21}H_{20}O_{11}$	Flavonoids	522-12-3	1.31	2.37	up
D-Threose	$C_4H_8O_4$	Saccharides	95-43-2	1.35	2.06	up
1,7-Dimethylxanthine	$C_7H_8N_4O_2$	Nucleotides	611-59-6	1.34	1.99	up
Quinic Acid	$C_7H_{12}O_6$	Organic acids	77-95-2	1.33	-1.44	down
8-Hydroxy-2-deoxyguanosine	$C_{10}H_{13}N_5O_5$	Nucleotides	88847-89-6	1.28	-1.54	down
N-Acetyl-L-Aspartic Acid	$C_6H_9NO_5$	Amino acids	997-55-7	1.34	-1.58	down
Pantetheine	$C_{11}H_{22}N_2O_4S$	Alkaloids	496-65-1	1.34	-1.64	down
4-Hydroxyhippurate	$C_9H_9NO_4$	Amino acids	2482-25-9	1.24	-1.99	down
LysoPC 22:6	$C_{30}H_{50}NO_7P$	Lipids	/	1.35	-2.07	down
L-Alanyl-L-Phenylalanine	$C_{12}H_{16}N_2O_3$	Amino acids	3061-90-3	1.34	-2.12	down
Bavachin	$C_{20}H_{20}O_4$	Flavonoids	19879-32-4	1.34	-2.48	down
Glutathione reduced form	$C_{10}H_{17}N_3O_6S$	Amino acids	70-18-8	1.34	-2.84	down
Abbeokutone	$C_{20}H_{32}O_3$	Others	16836-28-5	1.35	-12.60	down

VIP denotes the weight value of the variable from the OPLS-DA model. Generally, the metabolites of VIP > 1 are considered as DAMs. log_2(FC): the ratio of the average expression of metabolites in the two groups of samples. Positive value means up-regulation; negative value means down-regulation FC fold change. / denotes none corresponding number.
